# Androgen receptor signalling in the male adrenal facilitates X-zone regression, cell turnover and protects against adrenal degeneration during ageing

**DOI:** 10.1038/s41598-019-46049-3

**Published:** 2019-07-18

**Authors:** Anne-Louise Gannon, Laura O’Hara, J. Ian Mason, Anne Jørgensen, Hanne Frederiksen, Laura Milne, Sarah Smith, Rod T. Mitchell, Lee B. Smith

**Affiliations:** 1 MRC Centre for Reproductive Health, University of Edinburgh, The Queen’s Medical Research Institute, 47 Little France Crescent, Edinburgh, EH16 4TJ UK; 2 Centre for Discovery Brain Sciences, Hugh Robson Building, George Square, Edinburgh, EH8 9XD UK; 3 Department of Growth and Reproduction, Rigshospitalet, University of Copenhagen, Copenhagen, Denmark; 4 International Centre for Research and Research Training in Endocrine Disruption of Male Reproduction and Child Health (EDMaRC), Rigshospitalet, Copenhagen, Denmark; 5 Edinburgh Genome Foundry, Michael Swann Building, Max Bonn Crescent, Edinburgh, EH9 3BF UK; 6 School of Environmental and Life Sciences, Faculty of Science, University of Newcastle, Callaghan, 2308 NSW Australia

**Keywords:** Adrenal gland diseases, Adrenal tumours

## Abstract

Androgens are known to be an essential regulator of male health. Androgen receptor (AR) is widely expressed throughout the adrenal cortex, yet the wider role for androgen signalling in the adrenal remains underexplored. To investigate AR-dependent and AR-independent androgen signalling in the adrenal, we used a novel mouse model with a specific ablation of androgen receptor in the adrenal cortex with or without reduction of circulating androgen levels by castration. Our results describe AR expression in the human and mouse adrenal and highlight that the mouse is a viable model to investigate androgen signalling in the adrenal cortex. We show androgen signalling via AR is required for X-zone regression during puberty. Furthermore, cortex measurements define differences in X-zone morphology depending on whether circulating androgens or AR have been removed. We show androgens promote both cortical cell differentiation and apoptosis but are dispensable for the formation of the definitive cortex. Additionally, investigation of aged mice with AR ablation reveals severe cortex disruption, spindle cell hyperplasia and X-zone expansion. The data described herein demonstrates AR-signalling is required to facilitate X-zone regression, cell clearance and to protect against adrenal degeneration during ageing.

## Introduction

Androgens are essential regulators of male health, primarily in the maintenance and development of male sexual characteristics^1^. A decline in circulating androgens has been associated with co-morbidities such as obesity^2^ cardiac disease^3^ and metabolic syndrome^4^. Androgen production in men occurs predominantly in the testis (~90%) with a small proportion produced in the zona reticularis (ZR) of the adrenal cortex^5,6^. Androgens primarily exert their effects through their cognate androgen receptor (AR), a nuclear receptor to testosterone and dihydrotestosterone. Upon binding, AR undergoes translocation into the nucleus to regulate gene transcription^7^. Androgens and AR have also been shown to act independently of each other to regulate cellular processes^8^.

Previous research has focussed upon the body-wide impact of adrenal androgens^9^. However, whilst AR is abundantly expressed in the adrenal cortex of both rodents^10,11^, and humans^12,13^, surprisingly little is known about androgen action on the adrenal cortex itself. This gap in our understanding is at least in part due to the perceived lack of suitable animal models. Mice have largely been overlooked as a model system for the human adrenal as mouse adrenals are unable to produce androgens due to the silencing of *Cyp17a1* early in development. This lack of expression results in no 17α hydroxylase and 17, 20 lyse activity and consequently the rodent adrenal does not have a zona reticularis^14,15^. The fetal adrenal is thought to give rise to the adult adrenal cortex in both humans and mice^16,17^, so understanding the mechanisms that underpin its regulation is essential. In the mouse, these fetal cells are maintained for a period postnatally and regress in a sex dependent manner. In the human, this zone is known as the ‘fetal zone’, with the mouse homologue termed the ‘X-zone’^18,19^. Historical studies using castrated mice show that removal of circulating androgens leads to the redevelopment of an additional cortex zone known as the transient X-zone^20^. The redevelopment of the X-zone following castration and the abundant expression of AR in the mouse adrenal suggests that androgens are important regulators of the adrenal cortex and that despite not producing adrenal androgens, the mouse has utility for dissecting the role of androgen signalling within the adrenal. Previous global knockouts have ablated AR from the adrenal^21^, however, the caveats of this, like many global knockout models, is that the adrenals both produce and respond to multiple endocrine stimuli. For these reasons, it is difficult to attribute any phenotype to perturbed endocrine signalling locally rather than disruption of the wider hypothalamic-pituitary-axis.

For these reasons, we generated an adrenal-specific androgen receptor knockout model (Ad-ARKO) that ablates AR from the adrenal cortex in fetal and adult life^22^, permitting investigation of adrenal AR signalling. Due to the ability of AR and androgens to act independently of each other, additional models of castrated wild-type and castrated AR-ablated animals were investigated in parallel with the Ad-ARKO model, allowing us to independently dissect both androgen and AR action in the adrenal. We show that androgen signalling via the androgen receptor required for X-zone regression, furthermore, that androgens and androgen receptor also work independently to regulate multiple aspects of adrenal cortex function and morphology.

## Results

### Androgen receptor is widely expressed throughout the human and mouse adrenal cortex

**Figure 1:**
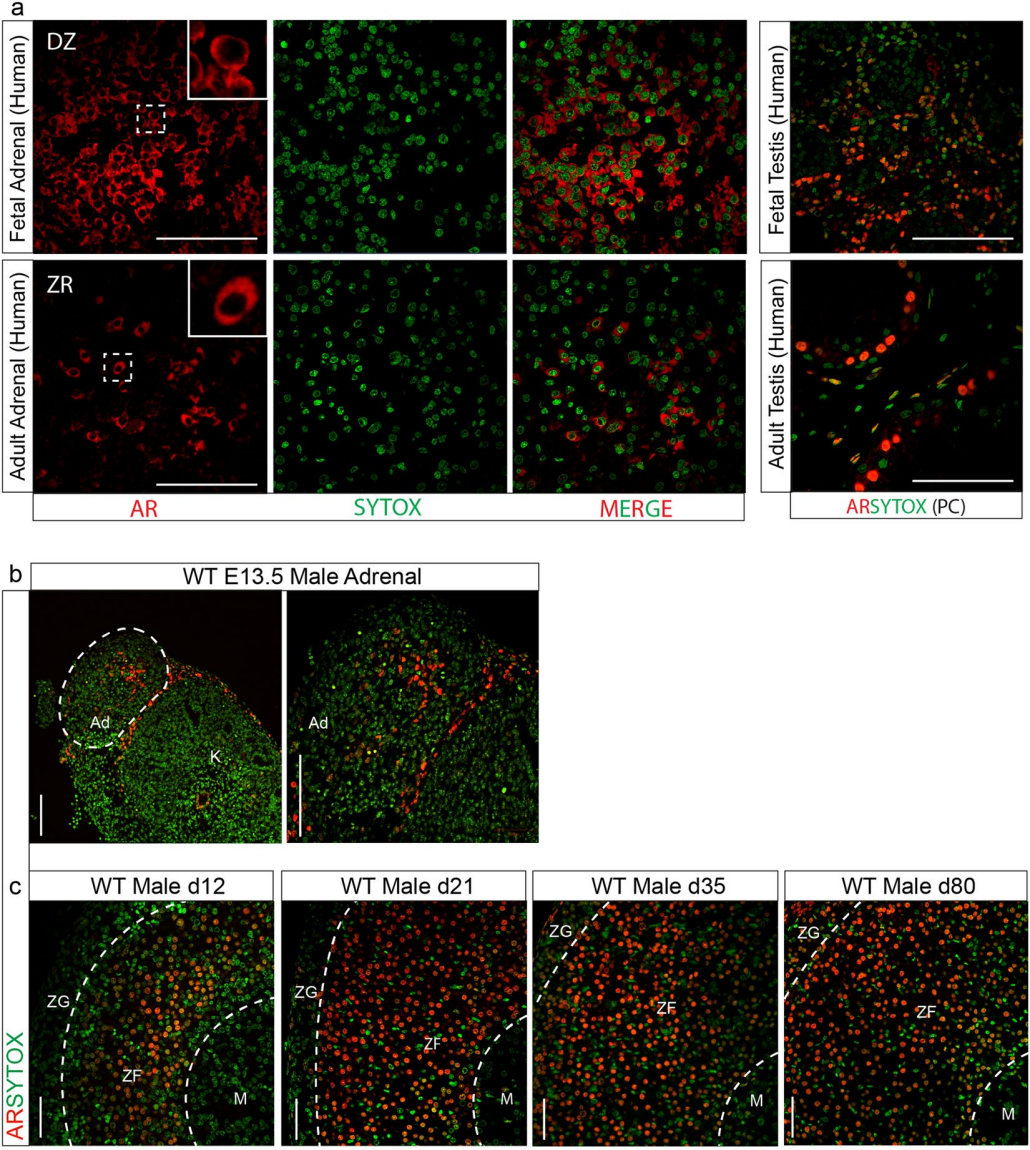
Androgen receptor expression in the adrenal cortex. (**a**) AR immunostaining details AR expression in the cytoplasm of the definitive zone in fetal (19 weeks) and in the cytoplasm of the zona reticularis in adult human adrenals (N = 2). Human fetal and adult adrenal samples are male. (**b**) AR localisation can be detected at e13.5 in mouse embryonic adrenals, with staining in the central region of the fetal adrenal (N = 3). (**c**) Expression of AR observed during development shows changes in localisation from the inner most cortex/medulla boundary at d12 to be present in the ZF at d21 and d35 and at d80 AR can be seen in the ZF and ZG (N = 5) (red = androgen receptor, green = sytox counterstain). PC = positive control, DZ = Definitive zone, ZR = Zona reticularis M = Medulla, ZF = Zona fasciculata, ZG = Zona glomerulosa. Scale bars 100 µm.

AR mRNA has been shown to be present in human and mouse adrenals^12,23^, however AR localisation in the adrenal cortex in human and mice has yet to be defined. To establish AR location within the adrenal cortex, human and mouse adrenals were examined using immunohistochemistry. Expression of human AR can be seen in the definitive cortex in fetal adrenals and in the zona reticularis of the adult adrenal. Unexpectedly, despite AR being a nuclear receptor, localisation of AR in human tissue is observed in the cytoplasm (Fig. 1a). Analysis of the mouse fetal adrenal shows nuclear AR protein expression throughout the adrenal at (e)13.5 (Fig. 1b). AR protein is also present in the postnatal male mouse adrenal when examined at d12, d21, d35 and d80. AR is localised in cells at the cortex-medulla boundary at d12; by d21 AR is localised in cells throughout the zona fasciculata (ZF), and from d35 onward AR can be seen in cells in the ZF and zona glomerulosa (ZG) (Fig. 1c). These data confirm the expression of AR in both human and mouse adrenals during development and in adulthood, although differing in cellular location in the human and mouse, this suggests that despite a lack of androgen production, the mouse adrenal has human relevance as a model system for the study of the within-adrenal response to androgen stimulation. For these reasons, we generated an adrenal-specific mouse model to investigate the role of androgen receptor in the adrenal cortex.

### Confirmation of ablation of androgen receptor from the mouse adrenal

**Figure 2:**
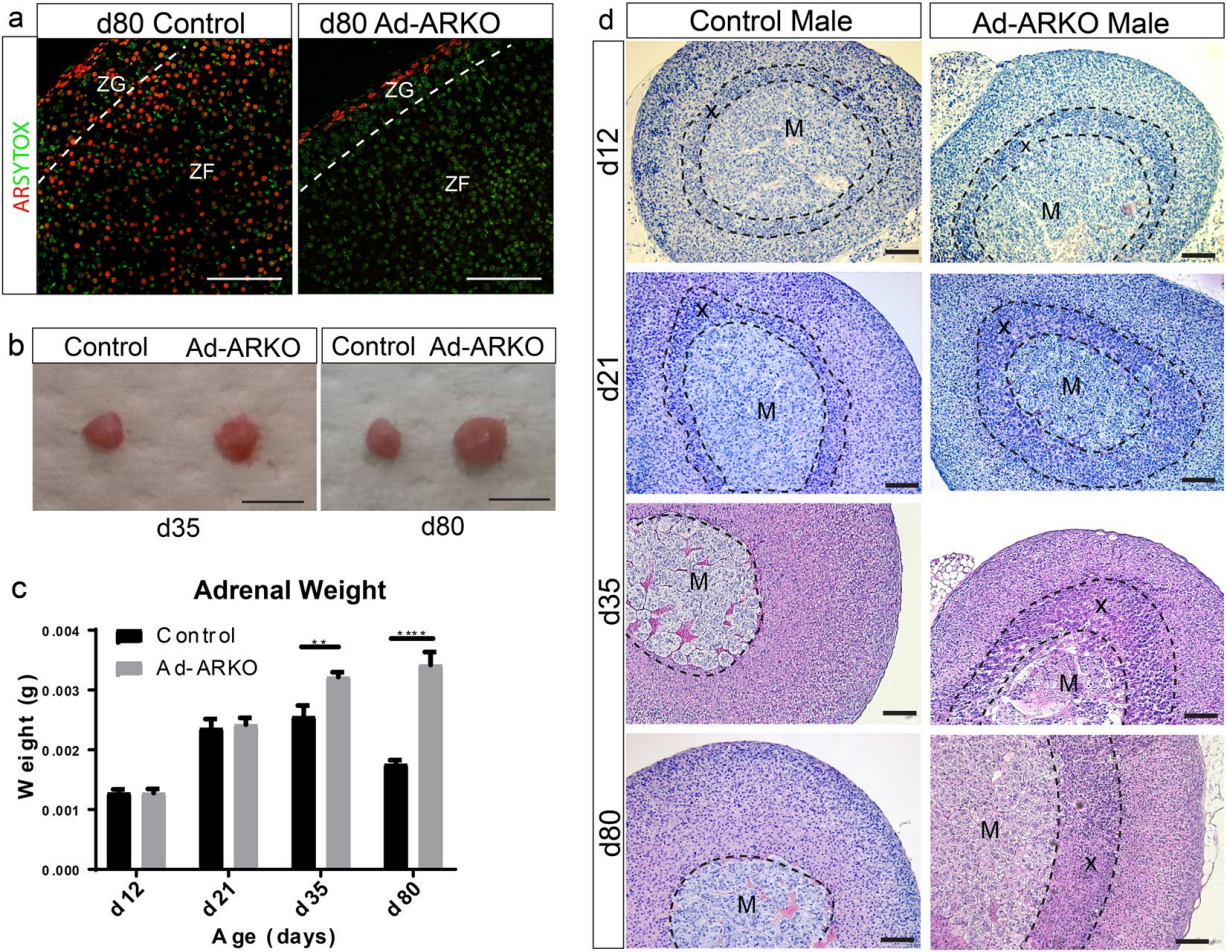
Ad-ARKO mice show increased adrenal weight and size and have an additional cortical zone that persists into adulthood. (**a**) Immunofluorescence staining confirms ablation of AR from adrenal cortex (red = androgen receptor, green = sytox counterstain). (**b**) Observations of whole tissue at d35 and d80 demonstrate Ad-ARKO males have larger adrenals (Scale bar 1 mm). (**c**) Weight analysis reveals Ad-ARKO males have significantly larger adrenals from d35, this difference in size is maintained into adulthood (Two-Way ANOVA; **p < *0*.*001*, n = 5, ****p < *0*.*0001*, n = 5, Tukey’s post-hoc analysis, error bars SEM) (**d**) Adrenal histology in d12 and d21 mice appear morphologically normal compared to control males. Full regression of the X-zone is usually complete by d35 which is demonstrated in controls, however there is an additional inner cortical zone still present in Ad-ARKO males at this time point. This inner cortical zone can again be observed in d80 males, with this extra cortical zone occupying a large proportion of the adrenal cortex. M = Medulla, X = X-zone, ZF = Zona fasciculata, ZG = Zona glomerulosa. Scale bars 50 µm.

We have previously described a novel method to ablate target genes from steroidogenic cell types through the development of a GFP-Cre-GC targeted to the mouse *Cyp11a1* locus to drive Cre Recombinase expression^22^. We established that this Cre line targets less than 20% of testicular Leydig cells^22^ and that loss of AR from Leydig cells has no discernible impact on circulating testosterone levels^24^. However, the GFP-Cre-GC line targets 100% of adrenal cortical cells, and immunostaining for AR in Ad-ARKO mice confirmed complete ablation of AR protein throughout the adrenal cortex (Fig. 2a). Interestingly, the adrenal capsule does not express *Cyp11a1*, therefore, *Cyp11a1*-Cre does not target these cells. This results in AR expression still being maintained in the capsule^22,25^, an important internal positive control for downstream studies. Initial observations of body weight, testis weight, and anogenital distance (AGD) show no differences in Ad-ARKO mice when compared to controls (Supplementary Fig. 1A–C).

### Ad-ARKO mice have enlarged adrenals and failed X-zone regression

Postnatal day 9 to 35 is a crucial remodelling period for the adrenal. During this time, the definitive cortex forms and the X-zone regresses^26^. Following AR ablation, we observe a fully formed definitive cortex in Ad-ARKO mice, demonstrating AR is dispensable for adrenal and definitive cortex formation. However, in line with previous studies suggesting androgens are essential for regression of the X-zone, we see a retained X-zone in Ad-ARKO mice. This is confirmed through adrenal weight and morphology analysis. Ad-ARKO adrenal weights are similar to controls in d12 or d21 male mice, however, at d35 and d80 there is a significant increase in adrenal weight compared to controls (Fig. 2c). Examination of adrenal morphology reveals the presence of a visibly more defined X-zone in d12 and d21 male Ad-ARKO adrenals compared to controls. Analysis at d35 and d80 shows X-zone retention in Ad-ARKOs (Fig. 2d). These results demonstrate that AR is essential for X-zone suppression.

### Confirmation of X-zone cells in Ad-ARKO adult males

**Figure 3:**
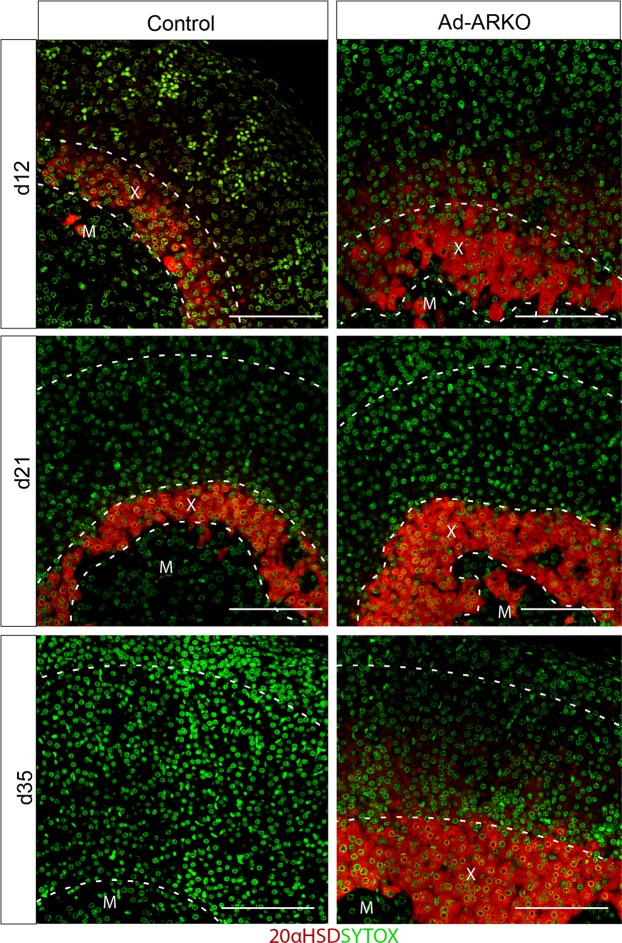
20 alpha-HSD demonstrates a more developed X-zone in Ad-ARKO mice. Protein localisation of 20 alpha-HSD (Red) in the developing postnatal adrenal. It can be seen that from d12 that the X-zone in Ad-ARKO mice is more developed compared to controls. M = Medulla, X = X-zone, ZF = Zona fasciculata, ZG = Zona glomerulosa. Scale bars 100 µm.

Due to the time period in which the mouse X-zone regresses, it has long been established that regulation of the adrenal by androgens does not occur until puberty^26^. Investigation of 20-α-hydroxysteroid dehydrogenase (20 alpha-HSD, produced by the mouse *Akr1c18* gene), an X-zone specific marker^27,28^, at d12, d21 and d35 shows that the X-zone in Ad-ARKO mice is more advanced in developmental stage than controls before, during and after puberty (Fig. 3). This challenges the current paradigm that suggests that androgen regulation of the adrenal cortex does not occur until later in postnatal development.

### Removal of circulating androgen results in X-zone development

**Figure 4:**
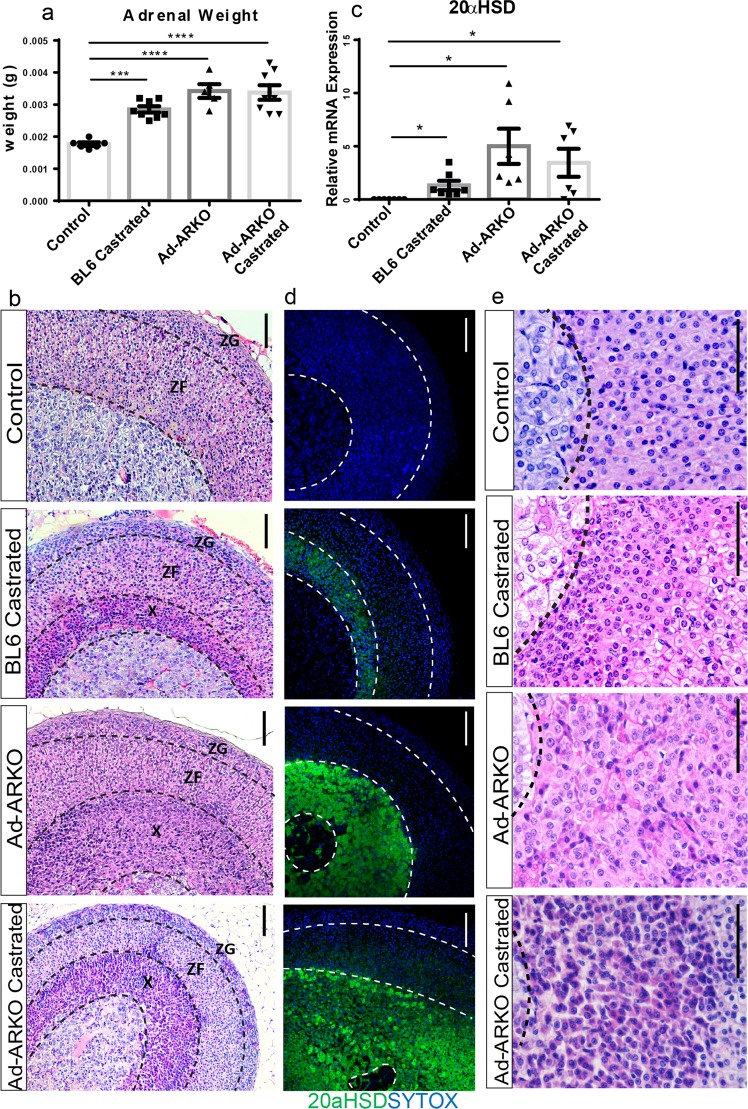
Analysis of additional androgen manipulation models. In all experimental cohorts an increase in weight is detected when compared to controls (one-way ANOVA; n = 8, ***p < *0*.*0001*, ****p < *0*.*0001*, Tukey’s post-hoc analysis, error bars SEM). (**b**) Morphology analysis confirms experimental cohorts have developed an additional cortical zone. (**c**) qRT-PCR data shows up regulation of 20 alpha HSD expression in castrated, Ad-ARKO and Ad-ARKO castrated mice (one-way ANOVA; n = 7–8, *p < *0*.*05*, Tukey’s post-hoc analysis, error bars SEM). (**d**) Immunostaining for 20 alpha-HSD confirms X-zone cells in adult cortex (Green = 20alpha-HSD, Blue = sytox counterstain). (**e**) Investigation of X-zones present in these cohorts show morphological differences in cell shape and size. All cohorts were collected at d80. M = Medulla, X = X-zone, ZF = Zona fasciculata, ZG = Zona glomerulosa. Scale bars 50 µm.

It is known that castration can induce development of an X-zone in males^20^, and that AR and androgens can work independently of each other^29–32^. To provide a comprehensive overview of androgen action in the adrenal, we included additional wild-type (WT) C57BL/6J (Bl6) ‘BL6 castrated’, and ‘Ad-ARKO castrated’, cohorts in our study to determine receptor and ligand-independent effects on adrenal regulation in young adult mice (d80). These additional cohorts also show a significant increase in adrenal weight compared to controls (Fig. 4a). Morphology and 20 alpha-HSD immunolocalisation analysis conforms X-zone re-development in Bl6 castrated, and a change in the morphology of the X-zone in Ad-ARKO castrated mice (Fig. 4b,c). Furthermore, we confirm the presence of AR in WT female, and WT castrated male X-zones (Supplementary Fig. 1D). Although all experimental cohorts display an X-zone, there are clear differences in the distribution of 20 alpha-HSD localization and X-zone morphology (Fig. 4e). This suggests that the X-zone and cortex could be impacted differently depending whether circulating androgens or AR have been ablated.

### Morphologies of X-zones in Bl6 castrated, Ad-ARKO and Ad-ARKO castrated show distinct characteristics

**Figure 5:**
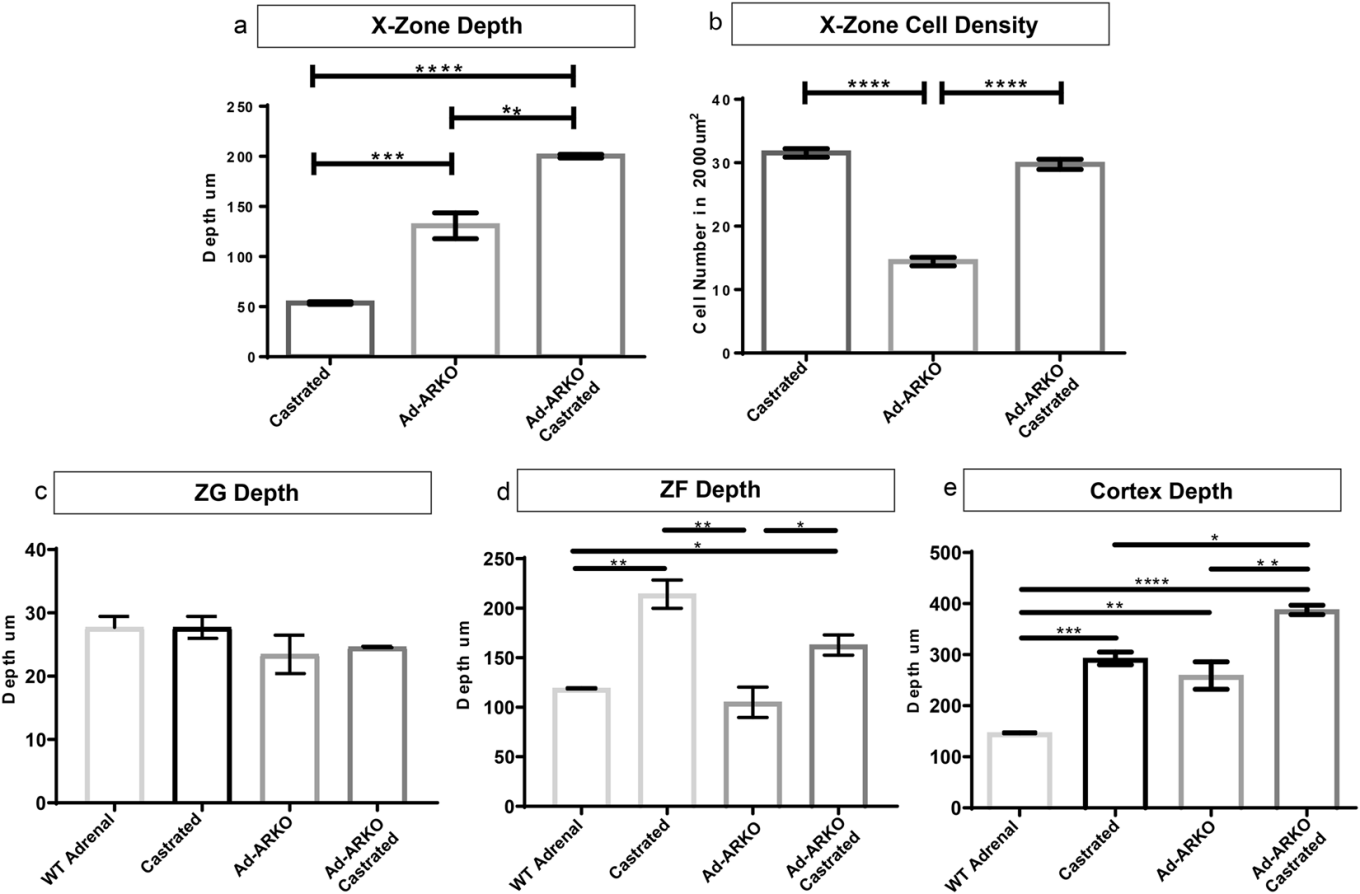
X-zones in BL6 Castrated, Ad-ARKO, and Ad-ARKO Castrated mice have distinct morphological differences. (**a**) Measurements of X-zone depth reveal that Ad-ARKO and Ad-ARKO Castrated mice have a significantly larger X-Zone than BL6 Castrated mice (one-way ANOVA; ***p < *0*.*0001*, ****p < *0*.*0001*, Tukey’s post-hoc analysis, error bars SEM). (**b**) The X-zone in BL6 Castrated mice is significantly denser than in Ad-ARKO mice, however upon castration of Ad-ARKO males, this cell density changes to be similar to Bl6 castrated mice (one-way ANOVA; ****p < *0*.*0001*, Tukey’s post-hoc analysis, error bars SEM). (**c**) There is no significant difference in ZG depth between the four cohorts. (**d**) A significantly smaller ZF depth is observed in WT male adrenals and Ad-ARKO mice compared to both castrated cohorts (one-way ANOVA; *p < *0*.*05*, **p < *0*.*001*, Tukey’s post-hoc analysis, error bars SEM). (**e**) All experimental cohorts have a significantly larger overall cortex size compared to WT male adrenals (one-way ANOVA; ****p < *0*.*0001*, ***p < *0*.*0001*, Tukey’s post-hoc analysis, error bars SEM). Ad-ARKO castrated mice have a larger overall cortex (ZG, ZF and X-zone) compared to Bl6 castrated and Ad-ARKO mice (one-way ANOVA; *p < *0*.*05*, **p < *0*.*001*, Tukey’s post-hoc analysis, error bars SEM). All cohorts were collected at d80.

Following the observation of differing X-zone morphologies after loss of AR or androgens, depth and cell density measurements were performed to distinguish differences in cortex composition between experimental cohorts. Measurements consisted of X-zone depth, X-zone cell density, ZG depth and ZF depth. X-zones in Ad-ARKO and Ad-ARKO castrated mice are significantly larger than Bl6 castrated mice (Fig. 5a). X-zones in Bl6 castrated and Ad-ARKO castrated have significantly denser X-zones per 2000 µm^2^ than Ad-ARKO mice (Fig. 5b). For analysis of cortex zones, a WT male adrenal has also been included for comparison. Measurements of the ZG reveals no changes in depth upon androgen manipulation between any cohorts (Fig. 5c). ZF depth is significantly smaller in Ad-ARKO mice compared to castrated cohorts however are similar in size to WT male adrenals (Fig. 5d). Measurements of complete cortex depth (ZG, ZF and X-zone) show all experimental cohorts have a larger overall cortex compared to WT male adrenals. A significant increase is also detected in Ad-ARKO castrated mice when compared to Bl6 castrated and Ad-ARKO mice (Fig. 5e). These data coupled with our morphological analysis suggest that AR and circulating androgens are able to act on the adrenal gland independently of each other, as well as in concert.

### Transcript analysis reveals changes in key adrenal regulators

**Figure 6:**
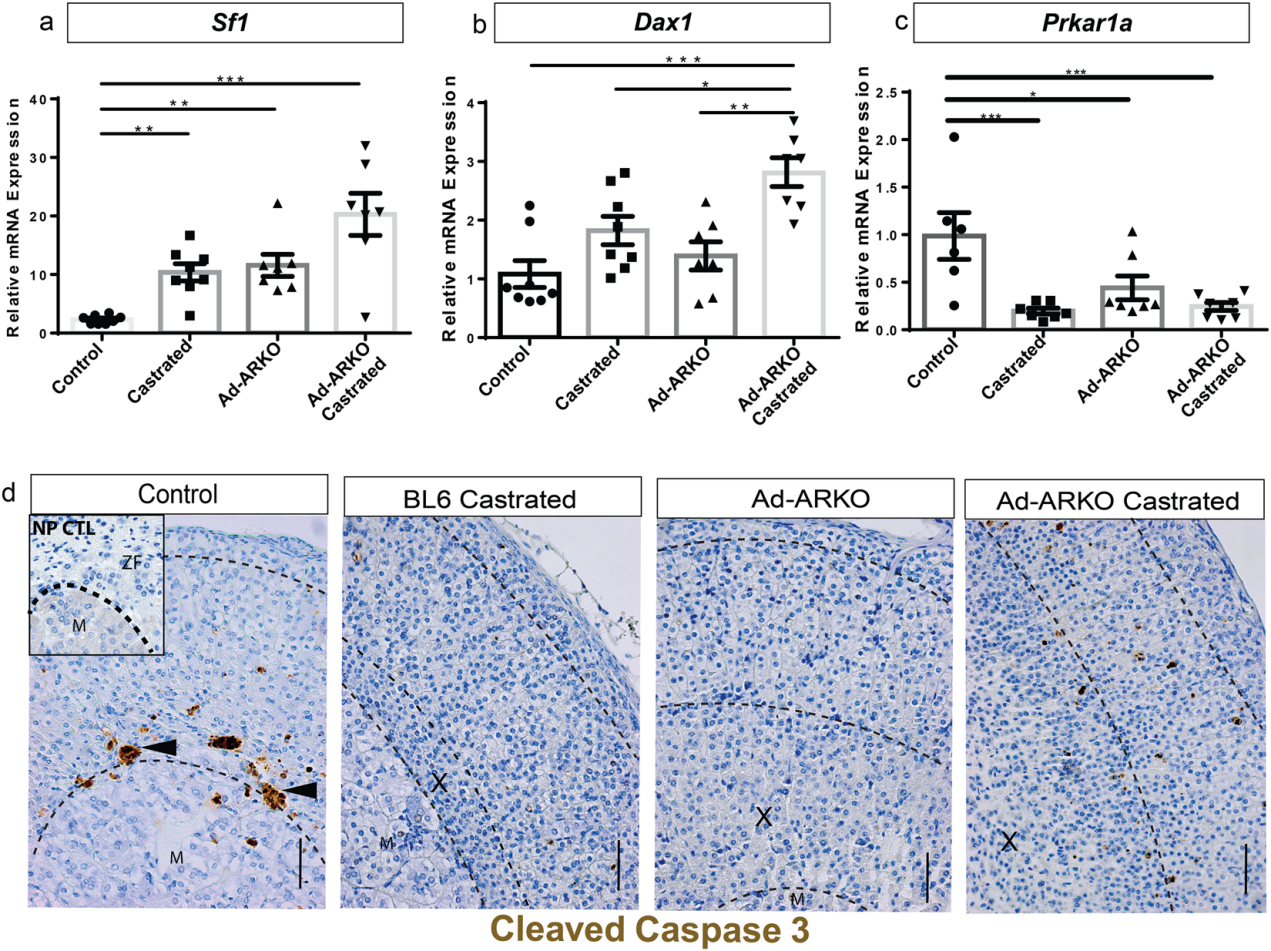
Loss of androgen signalling results in changes in key developmental genes. (**a**) *Sf1* gene expression analysis shows an increase in all experimental cohorts (one-way ANOVA; n = 8, **p < *0*.*001*, ***p < *0*.*0001*, Tukey’s post-hoc analysis, error bars SEM), however this increase was not observed in Ad-ARKO castrated males. (**b**) *Dax1* gene expression has increased in Ad-ARKO castrated males, however this is not observed in BL6 castrated or Ad-ARKO mice (one-way ANOVA; n = 8, ***p < *0*.*0001*, Tukey’s post-hoc analysis, error bars SEM). (**c**) *Prkar1* gene expression has decreased in all experimental cohorts compared to controls (one-way ANOVA; n = 8, *p < *0*.*05*, ***p < *0*.*0001*, Tukey’s post-hoc analysis, error bars SEM). (**d**) Cleaved caspase 3 immunostaining shows cell death at the cortex medulla boundary. Cleaved caspase 3 staining is not present in Bl6 castrated mice or Ad-ARKO animals. In Ad-ARKO castrated mice cell death can be seen throughout the cortex. All cohorts were collected at d80. M = Medulla, X = X-zone. Scale bars 50 µm.

Following the observation of fluctuations in cortex zone size, we wanted to ascertain if this results in changes of known adrenal and X-zone regulators. Two well described transcription factors that have been shown to be key regulators of adrenal development are the nuclear receptors, steroidogenic factor-1 (SF1, NR5A1, also known as Ad4BP) and *Dax1* (Dosage sensitive sex reversal, Adrenal hypoplasia congenita, critical region on the X chromosome, gene-1, NR0B1/AHC). Ablation or mutations in *Sf1* results in adrenal agenesis, and adrenal failure in *Dax1* ablation models, so determining if these are impacted following loss of androgen signalling is essential^33–35^. Furthermore a recent study has shown ablation of *Prkar1a* in aged mice results in the maintenance of an X-zone in adulthood in males and following a pregnancy in females^36^. Due to a similar phenotype being observed in Ad-ARKO mice, we investigated transcript levels of *Sf1*, *Dax1* and *Prkar1a*. Transcript analysis of *Sf1* shows a significant increase in all experimental cohorts when compared with controls at d80 (Fig. 6a). *Dax1*, shows no increase in Bl6 castrated or Ad-ARKO mice, however, there is a significant increase in transcript in Ad-ARKO castrated mice (Fig. 6b). Results show a significant downregulation of *Prkar1a* in all of our cohorts when compared with controls (Fig. 6c). In addition to the aforementioned key regulators, transcript analysis of *Ctnnb1*, *Gata6*, *Gli1* and *Wnt4*, additional known regulators of the adrenal were also investigated (Supplementary Table 3A). No changes are detected in these genes in Ad-ARKO mice compared to controls. Due to the variation in cortex zones, we also confirmed that there was no impact on *Actb* our house-keeping gene or influence from genotype (Supplementary Fig. 1E,F). Together these data demonstrate an important role for androgens during adrenal homeostasis and suggest that genes thought to be regulating the X-zone are likely to be direct or indirect downstream targets of AR signalling.

### Immunohistochemistry analysis reveals changes in cell death marker

Due to failed regression of X-zone cells from the cortex during puberty, we investigated whether there is any disruption to normal adrenal cell apoptosis via immunostaining of cleaved caspase 3. Immunostaining of cleaved caspase 3 shows that in control mice, cortical cells undergo apoptosis at the cortex-medulla boundary, conversely, BL6 castrated and Ad-ARKO mice show no cleaved caspase-positive cells. However, Ad-ARKO castrated mice show aberrant apoptosis throughout the cortex (Fig. 6d). However, we see no differences in PCNA localisation across these cohorts compared to controls (Supplementary Fig. 1G). These results suggest that, in the absence of androgens and/or AR, the adrenal fails to appropriately differentiate its cortex zones, in addition to disruption to cell clearance from the cortex.

### Disruption to AR signalling in the adrenal cortex does not impact the HPA-axis

**Figure 7:**
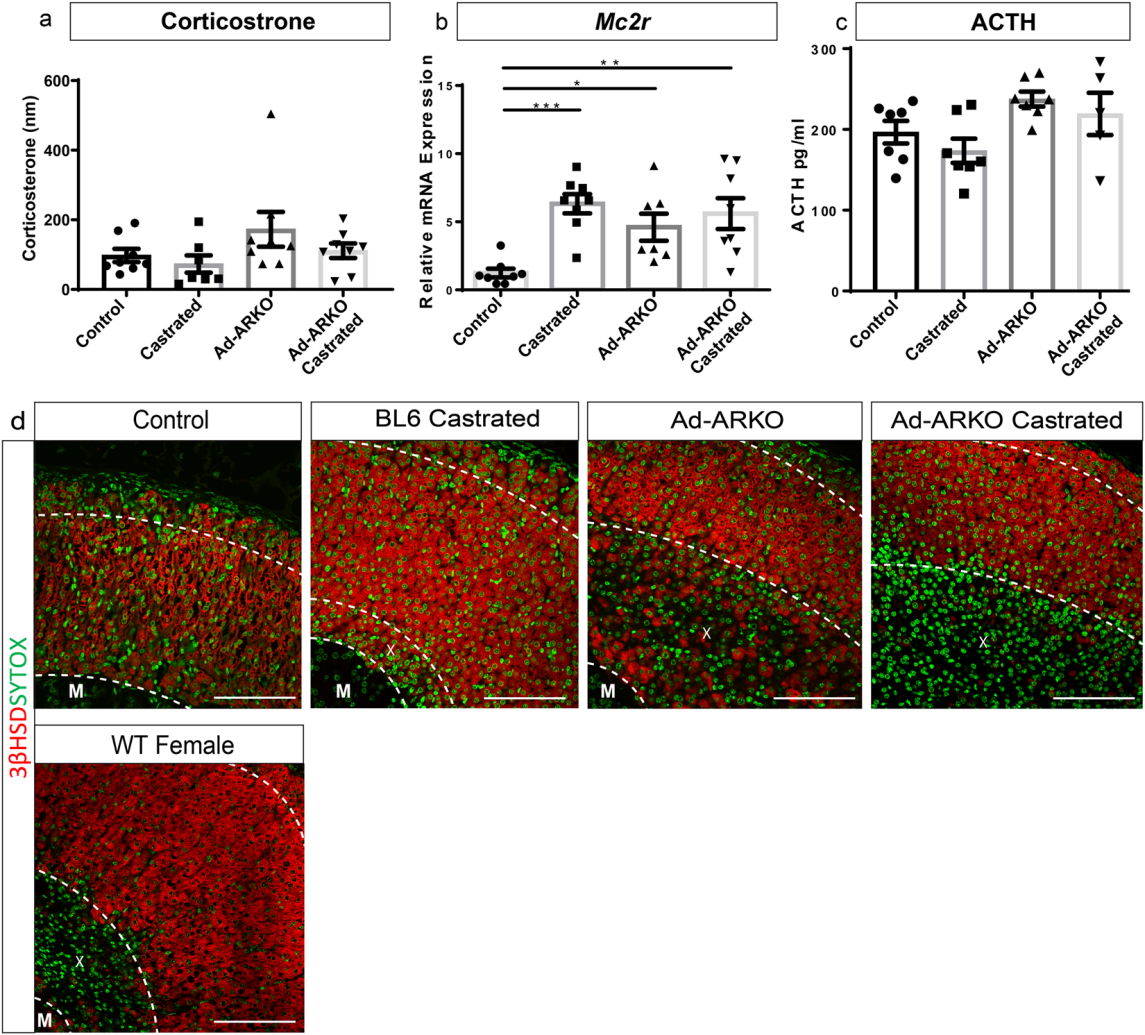
Disruption to androgen signalling does not affect circulating corticosterone levels. (**a**) Circulating corticosterone levels are unchanged in all experimental cohorts compared to controls at d80. (**b**) *Mc2r* gene expression shows an increase in all cohorts compared to controls (one-way ANOVA; n = 8, ***p < *0*.*0001*, *p < *0*.*05*, **p < *0*.*001*, Tukey’s post-hoc analysis, error bars SEM). (**c**) Circulating ACTH levels shows no changes in experimental cohorts compared to controls. (**d**) 3βHSD immunostaining (Red) shows the X-zone in castrated mice still positive for 3βHSD, however expression in Ad-ARKO X-zones is more sporadic with fewer positive cells and Ad-ARKO castrated mice show no expression in their X-zones. A 3βHSD negative X-zone is also noted in WT females. All cohorts were collected at d80. M = Medulla, X = X-zone. Scale bars 50 µm.

The maintenance of an X-zone, changes in adrenal regulators and changes in morphology following AR ablation would suggest and influence adrenal function. To assess this, we examined key biomarkers of the stress response pathway; circulating corticosterone concentration^37^, adrenal *Mc2r* expression^38^ and circulating pituitary ACTH concentrations^39^. Corticosterone analysis reveals no changes in any cohort compared with controls (Fig. 7a). We show a significant increase in *Mc2r* expression in all experimental cohorts compared to controls (Fig. 7b). However, consistent with no changes in corticosterone, circulating ACTH shows no changes in any cohort examined (Fig. 7c). These results reveal that HPA-axis control is independent of androgen signalling in these mice.

In addition to the analysis of stress hormones, mass spectrometry was used to analyse circulating androstenedione, testosterone, 17-OHP and progesterone in all cohorts (Supplementary Fig. 2A–D). The results show no changes in any cohort analysed when compared to controls. However, we do see fluctuations in several adrenal steroid enzymes and steroid receptors. Investigation of steroidogenesis markers revealed that in all experimental cohorts, CYP21A1 (protein essential for cholesterol conversion), is localised in all cortex zones compared to controls where protein is solely localised to the ZF^40^ (Supplementary Fig. 3A,B). This could lead to overactive steroidogenesis in the adrenal glands. Additionally, analysis of ZF marker AKR1B7 immunolocalisation revealed no positive cells in the adrenal cortex in Bl6 castrated and Ad-ARKO castrated mice. Interestingly, AKR1B7 is still expressed in Ad-ARKO mice (Supplementary Fig. 3C,D) suggesting androgens promote this expression via an AR-independent mechanism. We also noted a significant increase in glucocorticoid receptor (GR) in castrated cohorts, however, no changes in GR localisation was observed (Supplementary Fig. 3E,F). Furthermore, we also note a significant increase in AR in castrated mice (Supplementary Fig. 3G,H). We considered the possibility of AR directly regulating GR in the adrenal cortex, however, AR and GR are found in separate cell populations in the adrenal cortex, suggesting the results observed are independent or in-direct effects of AR-signalling (Supplementary Fig. 3I). A significant increase in expression of *Srd5a1* was detected in Ad-ARKO castrated mice, although not significant, we also see an increase in BL6 castrated mice. This increase is potentially in response to compensate for the removal of circulating androgens (Supplementary Fig. 3J).

Of particular interest, analysis of steroidogenesis marker 3β-Hydroxysteroid dehydrogenase (3β-HSD) reveals differential staining patterns in the X-zones of all experimental cohorts. BL6 castrated mice have 3β-HSD positive X-zones, suggesting these cells have come from a definitive cortex lineage. Ad-ARKO mice have sporadic 3β-HSD cells in their X-zone, however, following castration, no positive cells can be seen in the X-zone. This suggests that some cells in the X-zone of Ad-ARKO mice are steroidogenic and lose this capability following removal of androgens (Fig. 7d). This coincides with the denser X-zones of Ad-ARKO castrated mice noted in Fig. 5b. Wild-type (WT) females have 3β-HSD positive cells throughout their cortex but do not express 3β -HSD in their X-zone. This highlights that a WT X-zone in adulthood does not express 3β-HSD and suggests the presence of androgens without AR is driving X-zone cells down a steroidogenic cell lineage that is alleviated upon castration.

### Aged Ad-ARKO mice show increased disruption to the adrenal cortex

Despite X-zone maintenance, mice appear to be healthy with no major adverse phenotype, however, mice are analysed at d80 and are therefore still young. Due to the presence of an X-zone in all our experimental cohorts in addition to the downregulation of *Prkar1*, (Fig. 7d), we aged a cohort of Bl6 castrated, Ad-ARKO and Ad-ARKO castrated mice to 12 months (m). This is to determine if we observe a similar phenotype or additional disruption to the adrenal cortex following the prolonged loss of AR or circulating androgens.

**Figure 8:**
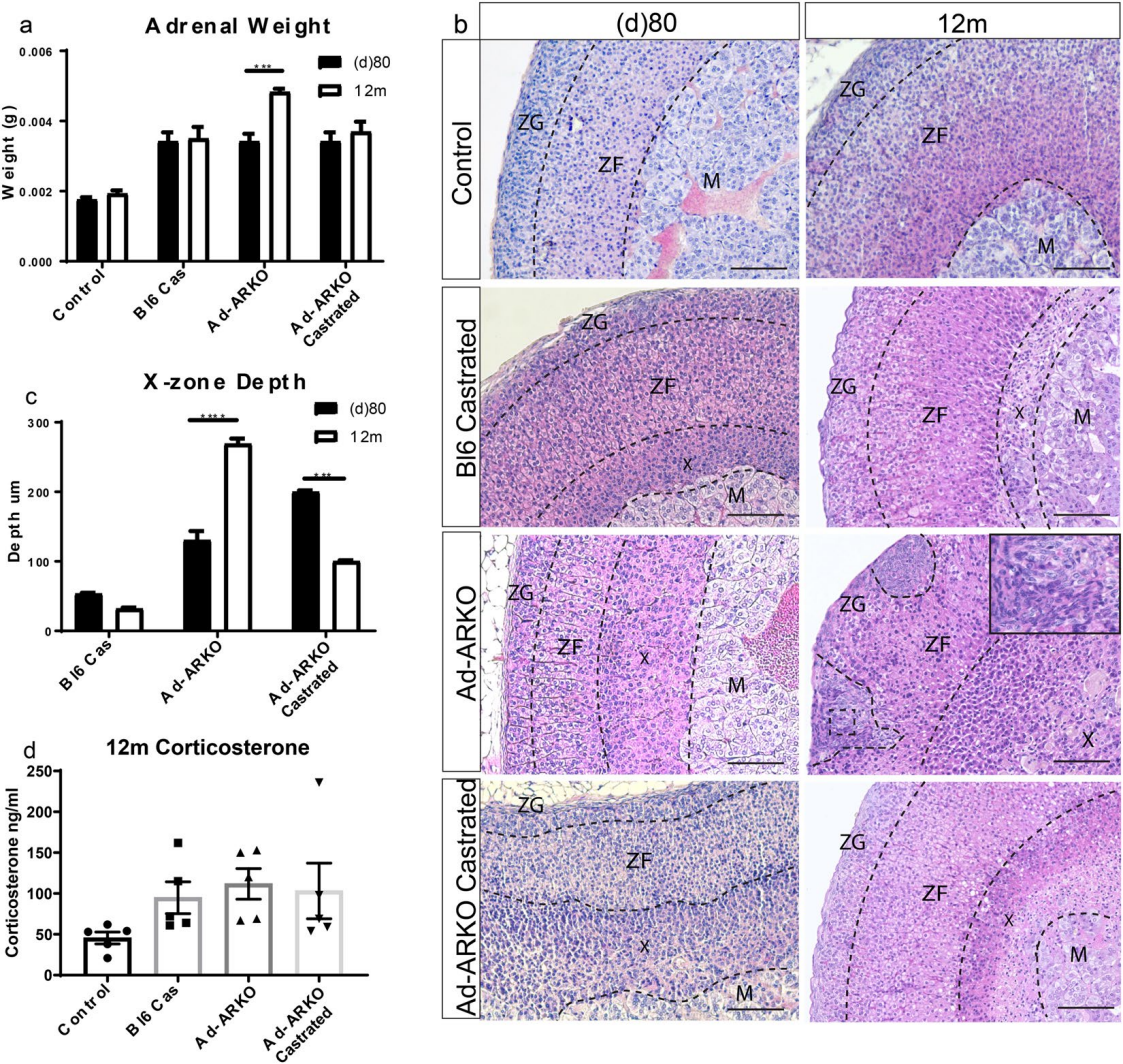
Aged Ad-ARKO mice display large cell clusters in capsule and large eosinophilic cells. (**a**) Adrenal weight analysis shows a significant increase in 12 m Ad-ARKO mice compared to d80 Ad-ARKO mice (two-way ANOVA; n = 5–8 ***p < *0*.*0001*, Tukey’s post-hoc analysis, error bars SEM), with no weight changes observed in any other cohort. (**b**) Morphology analysis at 12 m reveals that cohorts with no circulating androgens have no major cortex disruption, and no visible X-zone regression. 12 m Ad-ARKO mice have severe cortex disruption, with presence of large spindle cell lesions and enlarged X-zone. (**c**) Measurement of X-zone depth show a significant increase in 12 m Ad-ARKO mice compared to d80 Ad-ARKO mice (two-way ANOVA; n = 5–8 ****p < *0*.*0001*, Tukey’s post-hoc analysis, error bars SEM). In contrast, 12 m Ad-ARKO castrated mice show a significant decrease in X-Zone depth when compared to d80 Ad-ARKO mice (two-way ANOVA; n = 5–8 ****p < *0*.*0001*, Tukey’s post-hoc analysis, error bars SEM). (**d**) Circulating corticosterone analysis shows 12 m Ad-ARKO mice have no changes in corticosterone compared to 12 m WT controls. M = Medulla, X = X-zone, ZF = Zona fasciculata, ZG = Zona glomerulosa. Scale bars 50 µm.

Adrenal weight was unchanged in Bl6 castrated and Ad-ARKO castrated mice following the prolonged loss of circulating androgens, but a significant increase in adrenal weight is observed in 12 m Ad-ARKO mice compared to d80 Ad-ARKO mice (Fig. 8a). Consistent with this, analysis of adrenal morphology revealed no obvious visual difference in structure in 12 m WT controls when compared to d80 WT controls (Fig. 8b). Prolonged loss of circulating androgens in 12 m Bl6 castrated mice also show no major disruption to the adrenal cortex compared to d80 Bl6 castrated mice (Fig. 8b). Likewise, analysis of 12 m Ad-ARKO castrated mice show no disruption to the ZG and ZF regions of the adrenal cortex compared to d80 Ad-ARKO castrated mice (Fig. 8b). In addition, the X-zone in both these cohorts is significantly smaller than at d80 and is visibly breaking down (Fig. 8c). This phenotype is observed in aged virgin female mice, with eventual vacuolization of the X-zone^41^.

**Figure 9:**
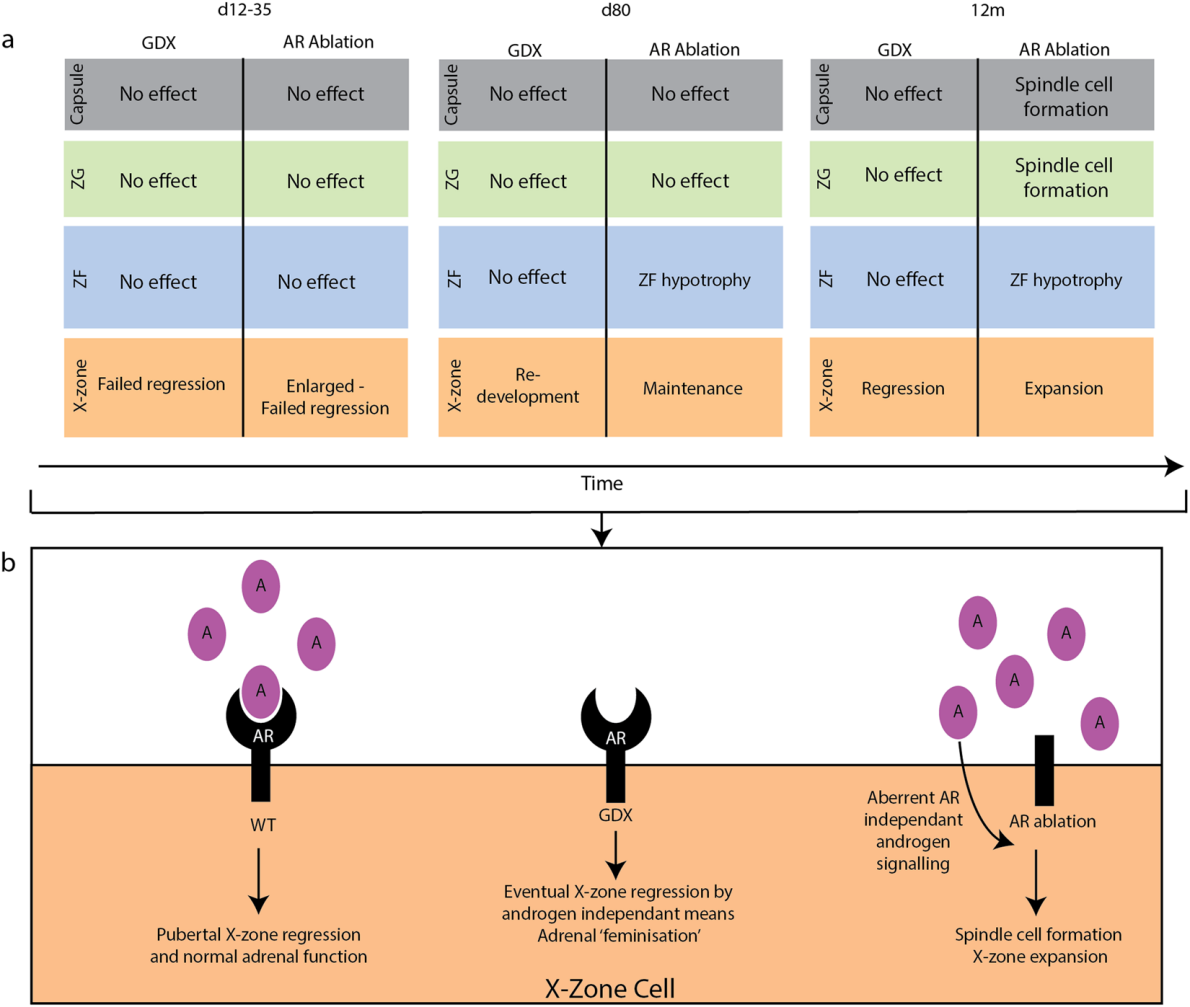
Diagram explaining the role of androgen receptor in the male adrenal cortex. (**a**) Summary of the differences described following loss of androgens or androgen receptor over the key time-points investigated in this study. Our data demonstrates over time, the loss of AR but not androgens results in spindle cell formation and X-zone expansion. (**b**) Diagram describing the role of androgens in X-zone cells. Androgens binding to AR promote X-zone regression and normal adrenal zonation in WT male mice. GDX results in the development of an X-zone with regression in aged male mice, phenocopying what is observed in the aging female. Loss of AR but not androgens results in adrenal degeneration in ageing male mice with aberrant AR independent androgen signalling driving X-zone expansion and differentiation.

In accordance with the increase in adrenal weight, severe cortex disruption is observed in 12 m Ad-ARKO animals compared to d80 Ad-ARKO mice (Fig. 8b). Large spindle cell lesions can be observed in the adrenal capsule, that extend down into the ZF. Additionally, these mice have an X-zone that is significantly larger than the X-zone that is observed in d80 Ad-ARKO mice (Fig. 8c). Consistent with d80 cohorts, no changes in corticosterone are observed in aged cohorts compared to controls (Fig. 8d). Together these data show that, whilst chronic loss of androgens does not exacerbate the phenotype established earlier in life, continued AR signalling is essential to prevent age-related degeneration of the adrenal cortex associated with further X-zone expansion and the development of spindle cell lesions. A summary diagram highlights these findings and the differences observed between androgen and androgen receptor loss in the adrenal cortex (Fig. 9a,b).

## Discussion

To examine the role of androgen action in the adrenal, we utilised a novel mouse model with a specific ablation of androgen receptor in the adrenal cortex compounded with additional reduction of circulating androgen levels by castration. Our results describe AR expression in the human and mouse adrenal suggesting the mouse has utility as a model system with which to investigate androgen signalling in the adrenal cortex. AR ablation does not disrupt the formation of the definitive cortex and does not result in any adverse sexual or behavioural phenotypes, however, androgen signalling via AR is required for X-zone regression in males during puberty. Cortex measurements defined differences in X-zone morphology depending on whether circulating androgens or adrenal cortex AR have been targeted, suggesting the presence of a more complex signalling network linked to androgens. Prolonged loss of circulating androgens in aged Bl6 castrated and Ad-ARKO castrated revealed X-zone regression, in a manner that phenocopies previous observations in ageing female adrenals^41^. However, aged males with AR ablation alone revealed severe cortex disruption, spindle cell hyperplasia and X-zone expansion. The data described herein demonstrates AR-signalling is integral for adrenal cortex function throughout life.

The current paradigm regarding X-zone regression suggests that the surge in testosterone at puberty drives X-zone involution^42^. However, our investigation of morphology and 20 alpha-HSD expression in Ad-ARKO mice adrenals demonstrates that X-zone cells are already more developed in size and abundance compared to controls at d12, d21 and d35. This tells us, contrary to the literature, that the negative regulation of the X-zone by androgens or androgen receptor is occurring prior to puberty. Although not the focus of the study, Hershkovitz *et al*. noted a decrease in adrenal 20 alpha-HSD activity prior to the increase in testosterone that occurs during puberty in male mice^28^. This fits with our hypothesis that androgen signalling is regulating X-zone cells prior to the peak in testosterone. However, due to the minimal amount of circulating androgens at this time point, it would appear that AR is able to regulate the X-zone with low levels of circulating androgens or independently of androgens during adrenal postnatal development. In recent years, there has been a drive to identify the molecular mechanisms that govern the switch between fetal/adult adrenal cells and the pathways involved in adrenal cortex remodelling. Zubair *et al*. demonstrated via lineage tracing studies that AD4BP/SF-1 (64) is essential for adrenal formation, confirming that understanding the mechanisms that control the X-zone is essential to understanding adrenal regulation. Furthermore, they hypothesised that there is still an unidentified adult adrenal enhancer that activates during a key developmental stage to switch development from fetal to adult cells. The challenge surrounding understanding X-zone control is undoubtedly due to a number of genes identified as potential regulators. A study conducted by Sahut-Bernola *et al*. showed that the X-zone remains when *Prkar1a* is ablated from the adrenal cortex^36^. Consistent with their model, we note that Ad-ARKO mice also have X-zone maintenance that expands through the cortex with age and development of lesions in the outer cortex. However, we do not see the development of Cushing’s syndrome nor the switching on of *Cyp17a1* in the adrenal cortex. Interestingly, we do note a significant downregulation in the transcript of *Prkar1a*. This suggests a relationship between AR and Protein Kinase A (PKA)^43^. The same group has recently extended these studies to look at castration and DHT treatment in their *Prkar1a* ablation models and found that androgen signalling is able to inhibit the PKA pathway via the RSPO/WNT/β-catenin pathway in the adrenal^44^.

Ablation of *Dax1* in the adrenal cortex also results in maintenance of the X-zone^34^. It was for this reason that *Dax1* was thought to control the switch between fetal and adult cells. With a similar phenotype being observed in Ad-ARKO mice, demonstrates that the switch from adult to fetal cells is a complex mechanism and that *Dax1* could be involved in the same pathway as androgens and PKA to drive adult cortex cell differentiation and X-zone regression. Additionally, *Dax1* ablation results in eventual adrenal failure as the pool of adrenocortical progenitor cells cannot be replenished. This is not something that is observed in our Ad-ARKO or castration models suggesting that AR does not regulate definitive cortex differentiation. However, unlike the *Dax1* ablation model, our Ad-AKRO model does not target AR in the adrenal capsule^22^, which is essential for coordination of ZG to ZF transition and maintaining the pool of progenitor cells^45,46^, so the authors cannot comment on the rolf of AR in this cell population.

A significant upregulation in *Sf1* transcript in all experimental cohorts was also observed. It has previously been shown that androgens act as a repressor of *Sf1* (52), an important mechanism to regulate *Sf1* and its target genes (52). Sustained elevated expression of *Sf1* has been implicated in conditions such as childhood adrenocortical tumours (53). Whilst it is speculation at this stage, our data imply that perturbed androgen signalling could be a contributory factor in *Sf1*-associated adrenal disease, this requires further investigation. To determine the exact nature of the relationship between these signalling pathways, it would be pertinent to look at the androgen response elements in the promoter regions of these genes to determine if AR is directly upstream or if the changes observed are an off-target effect caused by general disruption to the adrenal cortex. The genes investigated in this study following AR ablation are by no means an exhaustive list of known cortex and X-zone regulators. We have in the first instance, focused on a sub set of key regulators, however, following the results of this study and demonstrating the importance of AR in the adrenal, it would be essential to investigate other known regulators. There is avenue for future research into the role of AR and their interactions with TRβ1^47^ shown to be important in X-zone maintenance but not development, and activin, shown to promote X-zone apoptosis^48,49^. Further study would also seek to examine cohorts castrated at additional time points. This study examined absence of circulating androgens from the point of castration onwards in acute (14 days) and chronic (6 months) androgen loss, post puberty. A potential caveat of this is that there is still the potential for stimuli from steroid hormones. This has been shown to occur through memorized histone modification and DNA methylation^50,51^. This may be particularly pertinent for long terms castrated mice that will have had circulating androgens for 6 months. Future work would seek to perform castration before the onset of puberty to determine is this could be a potential confounder.

The maintenance of the X-zone in adulthood in Ad-ARKO mice brings into question cell clearance from the adrenal cortex. Under normal conditions adrenocortical cells migrate towards the cortex-medulla boundary and undergo apoptosis^48^. Protein localisation of Cleaved caspase 3 showed no apoptotic cells in BL/6 castrated or Ad-ARKO mice, suggesting androgen signalling is important for cell clearance from the adrenal through apoptosis. This phenotype has been documented previously in a global AR knockout model, however, this was thought to be a result of the loss of AR signalling from the pituitary^21^, our results show that this is a within-adrenal role for androgen-signalling.

Sahut-Bernola *et al*. described an increase in severity in adrenal cortex disruption with age in *Prkar1a* knockout mice, showing X-zone expansion and the development of primary pigmented adrenocortical disease (PPNAD) with age^36^. Due to the overlapping phenotype, we hypothesised that the severity of the phenotype in our androgen manipulation models would progress with age. Again, we noted a disparity between our models depending on whether AR or androgens have been targeted. Both castrated cohorts show no major adverse phenotypes, there is no increase in corticosterone, cortex disruption or X-zone expansion. X-zone measurements show X-zone regression, phenocopying what is observed in an ageing female, this phenotype is well described in the literature^41,42,52–54^. Conversely, the Ad-ARKO shows X-zone expansion and spindle cell hyperplasia. This result is of particular interest as it demonstrates that AR but not androgens are needed to protect against age-related degeneration of the cortex. Furthermore, that androgens are potentially still able to stimulate the adrenal in Ad-ARKO mice and without the presence of AR to bind to, could result in the aberrant stimulation of off target pathways. AKR1B7 and 3βHSD staining and X-zone measurements taken in our d80 cohorts further strengthen this. Measurements of the X-zone demonstrated significant morphological and size differenced depending on whether, androgens or AR has been targeted. Furthermore, AKR1B7 localisation is still observed in Ad-ARKO mice compared to castrated cohorts with no AKR1B7 expression. Additionally, 3βHSD showed localisation in Ad-ARKO adrenals but not in either castrated cohorts. This suggests that again, without the presence of AR to bind to, there is activation of off target signalling pathways in the adrenal cortex that requires further investigation.

The presence of AR in both human and mouse adrenal suggests that the mouse could provide a suitable model to investigate human conditions, however, due to the differences in localisation of adrenal AR in the human and rodent this could potentially suggest that although present in both, could have varying roles. This would need to be taken into consideration in future studies regarding adrenal androgen signalling and would be an avenue for future research.

In conclusion, this study not only highlights many new roles for androgen signalling in the adrenal but shows that the mouse has utility as a model to explore androgen action within the adrenal, opening up a novel entry point towards further increasing our understanding of adrenal function. Through cortex measurements, we demonstrate androgens and AR can act independently of each other and show that AR in mouse and human display similar spatiotemporal expression. Furthermore, we demonstrate that androgens act via AR to regress the X-zone and influence both cortical cell differentiation and apoptosis. We highlight that AR is potentially upstream of key adrenal-developmental genes but is dispensable in the formation of the definitive cortex. Finally, we demonstrate that androgens acting via AR protects against age-related degeneration of the adrenal cortex, with implications for our understanding in preventing adrenal degeneration and tumour development.

## Methods

### Ethics statement

All mice used in experiments were under a strict standard of care and experimental planning covered by licensed approval from the UK Home Office (License Number 80/7704) and adhere to the ARRIVE (Animal Research: Reporting of *In Vivo* Experiments) guidelines^55^. Ethical approval was obtained for the use of archived human fetal adrenal tissue from the East Scotland Research Ethics Committee (Reference Number: LREC08/S1101/1). Ethical approval was obtained for the use of archived human adult adrenal tissue from the Danish regional ethics committee (Reference Number: H-1-2012-007).

### Targeted ablation of AR from the adrenal cortex using Cyp11a1-GC Cre

To specifically ablate AR from the adrenal cortex, Cre/*loxP* technology was used. Male C57BL/6 mice carrying a random insertion of the *Cyp11a1*-GC Cre^22^ were mated to C57BL/6 female mice homozygous for floxed AR^56^. Male offspring were either *Cyp11a1*^+/GC^; AR^fl/y^ mice with adrenal androgen receptor ablation termed ‘Ad-ARKO’ or *Cyp11a1*^+/+^; AR^fl/y^ ‘control’ littermates.

### PCR genotyping of mice

Mice were genotyped for the inheritance of Cre recombinase as previously described^57^. PCR amplification products were resolved using the QIAxcel capillary system (QIAGEN, Crawley, United Kingdom). An amplicon of 102 bp indicated the inheritance of the Cre recombinase transgene.

### Removal of circulating androgen through castration

Isoflurane was administered via inhalation. A single 1 cm incision was made into the scrotum and testes exposed and removed. Following removal of testes, the site of incision was closed with sterile sutures. Mice were injected subcutaneously with Buprenorphine 0.05 mg/kg, whilst anaesthetized, and allowed to recover whilst being monitored. Mice were closely monitored over 24 hours for any welfare problems, and twice daily from then onwards. At the end of the experiment, mice were culled by inhalation of CO_2_ until unconscious, followed by cervical dislocation. These animals were termed ‘BL6 castrated’ and ‘Ad-ARKO castrated’.

Two castration time points are used in this study, short term and long term. Short-term castrations were performed on postnatal day 64, allowed to age for 14 days and collected at postnatal day 80. Long-term castrations were performed on 6-month-old mice, left to age another 6 months and collected at 12 months old.

### Tissue collection and processing

Body weight was measured, and adrenals were removed and weighed. Reproductive organs were also examined, removed and weighed. Tissues were fixed in Bouin’s fixative (Clin-Tech, Guildford, UK) for 4 hours (adrenals). Bouin’s-fixed tissues were processed and embedded in paraffin wax, and 5 µm sections were used for histological analysis. Sections of adrenals were stained with haematoxylin and eosin using standard protocols and examined for histological abnormalities.

### Immunohistochemistry

Immunolocalization was performed either by a single antibody colourimetric (DAB) immunostaining method, as described previously^58^ a single or double antibody tyramide fluorescent immunostaining method, as described previously^22,59^, or automated Bond immunostaining method, as described previously^58^. Exceptions to this protocol are the H_2_O_2_ concentration washes performed at 3% H_2_O_2_ TBS. Antibodies used are listed in Table 1. A minimum of five individual sections for each age and genotype were immunostained in each experiment.

**Table 1:** Immunohistochemistry performed in this study, listing antibody source and method used.

Protein stained for	Method	Primary antibodies
HSD3B	Single fluorescence	HSD3B: Santa Cruz #sc30820
AR	Single fluorescence	AR: Spring Bioscience #M4070
AR-N20	DAB	Santa Cruz Biotechnology #sc-816
PCNA	DAB	Sigma #P-8825
AKR1B7	Single fluorescence	Santa Cruz Biotechnology #sc-27763
HSD20alpha	Single fluorescence	Aviva Systems Biology #OAGA00409
GR	DAB	Cell Signaling #12041
CYP21	DAB	Bioss antibodies #bs-2443R
CASP3	Bond	CASP3: Abcam #ab4051

### Quantitative RT-PCR

RNA was obtained from frozen adrenals from n = 8 d80 BL6 castrated, n = 8 Ad-ARKO, n = 8 Ad-ARKO castrated and n = 8 control mice, using the RNeasy Mini extraction kit with RNase-free DNase on the column digestion kit (Qiagen, Crawley, UK) according to the manufacturer’s protocol. RNA yield was quantified using a NanoDrop 1000 spectrophotometer (Thermo Fisher Scientific, Waltham, MA, USA). Random hexamer primed cDNA was prepared using the SuperScript VILO cDNA synthesis kit (Life Technologies) according to manufacturers’ protocols. Quantitative PCR was performed on d80 BL6 castrated, Ad-ARKO, Ad-ARKO castrated and control adrenals for the genes of interest listed in Table 2 using an ABI Prism 7900 Sequence Detection System (Applied Biosystems) and the Roche Universal Probe Library (Roche, Welwyn, UK). The expression of each gene was related to internal housekeeping gene assay *Actb* (Roche, Welwyn, UK).

**Table 2:** qPCR assays used, with sequences of primers and UPL probe numbers for each assay.

Transcript	5′ primer	3′ primer	Roche UPL probe
*Ar*	ttatgaagcagggatgactctg	gctgccagcattggagtt	12
*Nr3c1*	ccactgcaggagtctcacaa	gcaaagcatagcaggtttcc	91
*Cyp21a1*	ccaacctggatgagatggtt	ggattcttcccaggttccag	107
*Srd5a1*	gggaaactggatacaaaataccc	ccacgagctccccaaaata	41
*Hsd3b1*	gaactgcaggaggtcagagc	gcactgggcatccagaat	12
*Akr1c18*	tggccctagccaagagttt	gccaattggaaatcaaagacc	91
*Akr1b7*	ccaccttcgtggaactcag	cttggcctggggaagact	104
*Ctnnb1*	gcagcagcagtttgtgga	tgtggagagctccagtacacc	25
*Gli1*	ctgactgtgcccgagagtg	cgctgctgcaagaggact	84
*Dax1*	cgtgctctttaacccagacc	ccggatgtgctcagtaagg	3
*Sf1*	tccagtacggcaaggaaga	ccactgtgctcagctccac	18
*Prkar1a*	gctgaagtttacactgaggagga	cagccattgtcttataatcttttgg	16

### Extraction of steroid hormones from plasma

Immediately after culling, blood was collected from mice via cardiac puncture with a syringe and needle, blood was collected in EDTA coated tubes to prevent coagulation. Mice were collected at 10:00 am following minimal handling. Handling, culling and blood collection was performed in under two minutes. Plasma was separated by centrifugation and stored at −80.

### Quantification of hormone levels

Corticosterone in 12 m aged mice was measured using a mouse corticosterone ELISA kit (KO14-H5) according to manufacturer’s instructions. ACTH was measured using a mouse ACTH ELISA kit (ABIN415571) according to manufacturer’s instructions. All samples were run as a single assay for each hormone.

Serum analysis of d80 corticosterone and circulating androgens was achieved through the use of a new and sensitive isotope-dilution TurboFlow-LC-MS/MS method. Using this method, we are able to quantify androstenedione, testosterone, 17α-hydroxyprogesterone (17-OHP) progesterone, and corticosterone in human and mouse serum as previously described^60^, without modifications. However, since the steroid concentration in several of the mouse samples were higher than the normal analytical range for steroids in human serum, the linearity of calibration curves was investigated by preparing calibration materials in synthetic serum expanded in the high concentration area by including a total of 23 different dilutions of the standard stock solution. The linear range of calibration curves for regression analysis are shown in Supplementary Tables 1 and 2(A). All control material was based on different pools of human serum from children and adults, spiked in respectively low and high levels and not spiked serum pool. Samples were analyzed in 7 batches and each batch included standards for calibration curves, about 60 blind samples, one blank, three un-spiked serum pool samples, three pool controls spiked at respectively low and high levels. The inter-day variation, expressed as the relative standard deviation (RSD) was ≤8% for all analytes in both spike levels. The recovery was >89% for all analytes. Supplementary Tables 1 and 2(B) shows the limit of quantification^60^, the linear range for each individual calibration curves, and the inter-day validation. All chemical analyses were performed at the Dept. of Growth and Reproduction, Rigshospitalet, Copenhagen University Hospital.

### Quantification of cortex zones

Comparison of adrenal cortex zones was performed by serially sectioning the whole adrenal at 5 µm, which produced on average 50 slides, per one adrenal, with one section per slide. To account for the shape of the adrenal and allow for consistent measurements, counts were performed on slides 15, 25 and 35 in Ad-ARKO and BL6 castrated animals. X-Zone depth was measured from the medulla boundary to the outer edge of the X-zone, with 5 measurements per slide, across sections 15, 25 and 35 in 5 castrated, 5 Ad-ARKO and 5 Ad-ARKO castrated samples. X-zone depth was measured by placing a 5 × 5 square grid over an ×40 magnification image of the X-zone. Five 2000 µm squares were chosen at random and cells within each box were counted across sections 15, 25 and 35 in 5 castrated, 5 Ad-ARKO and 5 Ad-ARKO castrated samples. Zona Fasciculata/Zona Glomerulosa (ZF/ZG) depth was measured from the X-zone boundary to the capsule, with 5 measurements per slide, across sections 15, 25 and 35 in 5 castrated, 5 Ad-ARKO and 5 Ad-ARKO castrated samples. Overall capsule depth was measured from the medulla boundary to the capsule, with 5 measurements per slide, across sections 15, 25 and 35 in 5 castrated, 5 Ad-ARKO and 5 Ad-ARKO castrated samples. All individual counts can be seen in Supplementary Fig. 4A–C.

### Statistical analysis

Statistical analysis was performed using GraphPad Prism (version 7; GraphPad Software Inc., San Diego, CA, USA) using a two-tailed unpaired t-test (if comparing two groups), a one-way ANOVA with Tukey’s post-hoc test (if comparing multiple groups groups), or a two-way ANOVA with Tukey’s post-hoc test (if comparing multiple groups and variables) Values are expressed as means ± S.E.M.

## Supplementary information

Sup Figures 1-4 and Sup Tables 1-3

## Data Availability

All data generated as part of this study has been included in this manuscript in either the main body or in Supplementary Figures.
