# Supplementary material for: Androgen receptor signalling in the male adrenal facilitates X-zone regression, cell turnover and protects against adrenal degeneration during ageing
Source: Sci Rep. 2019 Jul 18;9:10457. doi: 10.1038/s41598-019-46049-3 (PMC6639311; doi:10.1038/s41598-019-46049-3)
Supplement: Supplementary file 1 — Sup Figures 1-4 and Sup Tables 1-3 [file 41598_2019_46049_MOESM1_ESM.pdf]

Androgen receptor signalling in the male adrenal facilitates X-zone regression, cell turnover and protects against adrenal degeneration during ageing

Anne-Louise Gannon[1, 5], Laura O'Hara[1, 2], J. Ian Mason[1], Anne Jørgensen[3], Hanne Frederiksen[3], Laura Milne[1, 4], Sarah Smith[1], Rod T. Mitchell[1] and Lee B. Smith[1, 5§]

[1] MRC Centre for Reproductive Health, University of Edinburgh, The Queen's Medical Research Institute, 47 Little France Crescent, Edinburgh, EH16 4TJ. UK. [2] Centre for Discovery Brain Sciences, Hugh Robson Building, George Square, Edinburgh, EH8 9XD, UK. [3] Department of Growth and Reproduction, Rigshospitalet, University of Copenhagen, Denmark, International Centre for Research and Research Training in Endocrine Disruption of Male Reproduction and Child Health (EDMaRC), Rigshospitalet, Denmark.

[4] Edinburgh Genome Foundry, Michael Swann Building, Max Bonn Crescent, Edinburgh, EH9 3BF. [5] School of Environmental and Life Sciences, Faculty of Science, University of Newcastle, Callaghan, 2308, NSW, Australia

§ To whom correspondence should be addressed: Professor Lee Smith, MRC Centre for Reproductive Health, University of Edinburgh, The Queen's Medical Research Institute, 47 Little France Crescent, Edinburgh EH16 4TJ, UK. Tel: +44 (0)131 242-9111 Email: Lee.Smith@ed.ac.uk

This work was funded by a Medical Research Council Program Grant Award (MR/N002970/1) (to LBS)

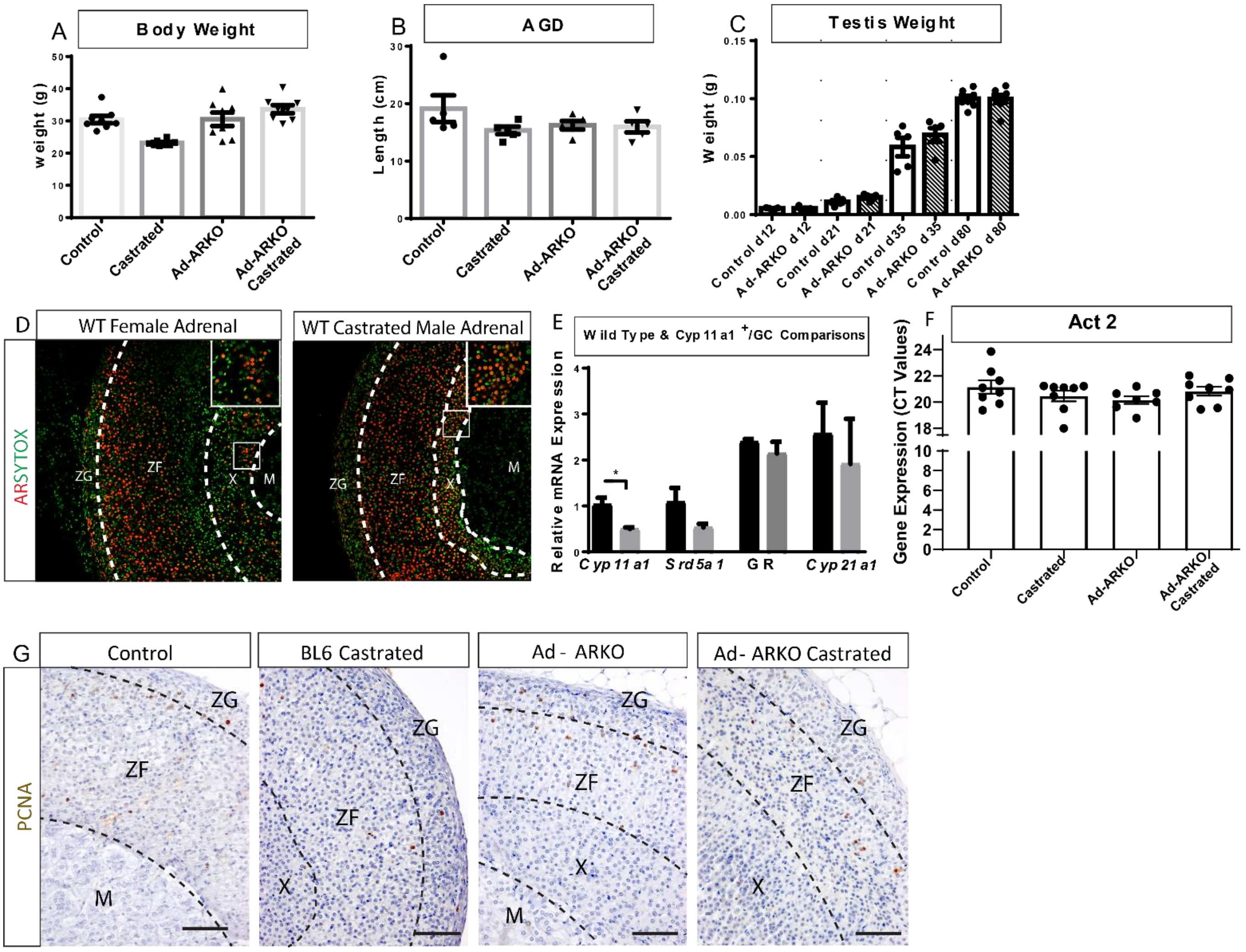

Supplementary Figure 1. Ad-ARKO mice show no differences in body weight, AGD or testis weight. (A) Body weight does not change in experimental cohorts when compared to controls at d80. (B) AGD remains unchanged in experimental cohorts when compared to controls at d80. (C) Testis weight throughout development does not change in Ad-ARKO mice when compared with controls. (D) Analysis of AR localisation in WT female adrenal and WT castrated male adrenals demonstrate positive AR cells in the X-zone. (E) Due to targeting of the Cre to the *Cyp11a1* locus, a decrease in *Cyp11a1* transcripts can be detected, however this does not influence any of our genes of interest as no significant difference can be observed between the genes in Cre+ and littermate controls at d80. (F) CT values were also examined to rule out influences of treatment on the housekeeping gene at d80. Results demonstrate less that a 1-fold variation between all cohorts. (G) PCNA immunostaining showed no observable differences in proliferation in any experimental cohort at d80. (J) AR localisation in the X-zone of d80 WT female adrenals and d80 WT castrated male adrenal. ZG= Zona Glomerulosa ZF= Zona Fasciculata, M=Medulla, X=X-zone. Scale Bars 50µm

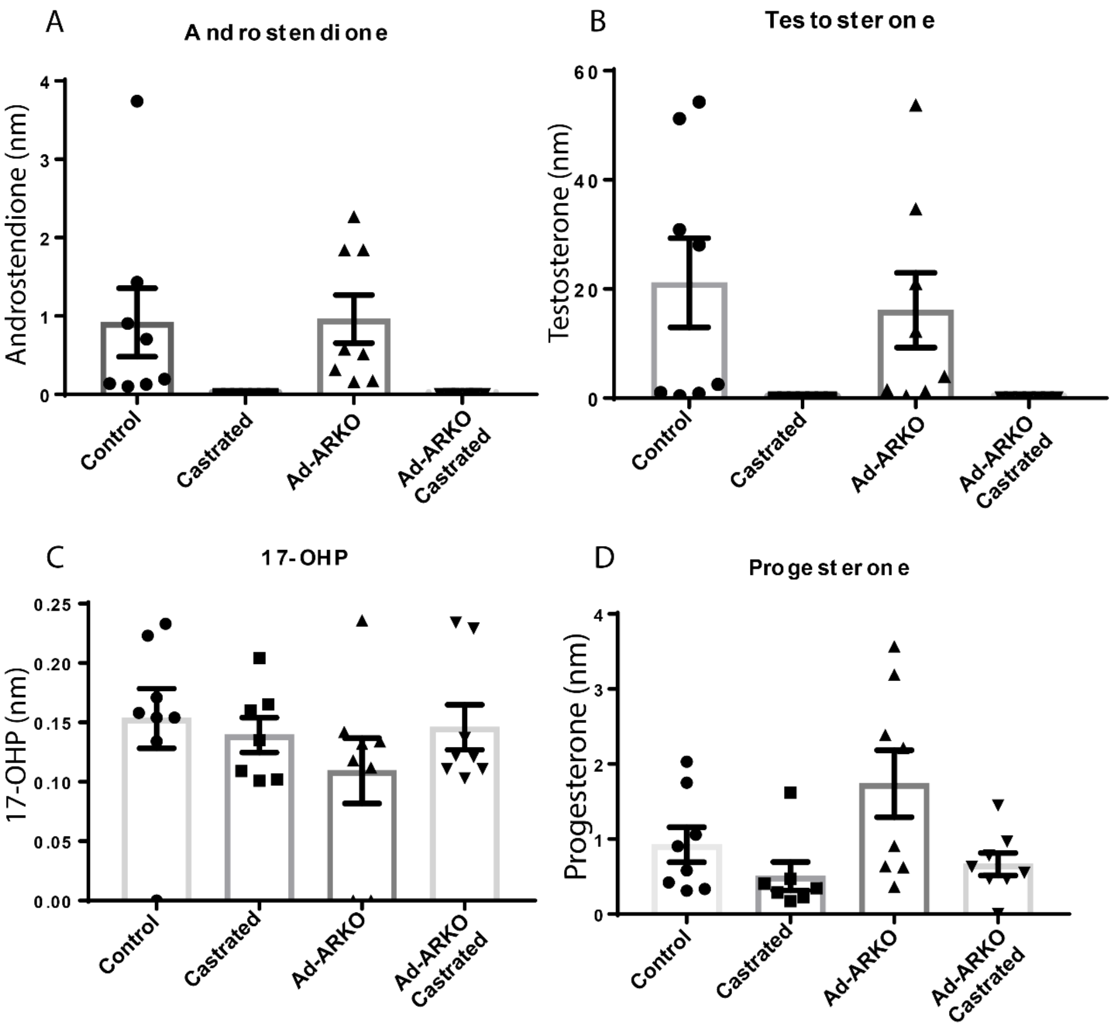

Supplementary Figure 2. Analysis of steroid metabolites revealed no changes in Ad-ARKO mice. Serum analysis via mass spectrometry revealed no changes in Androstenedione (A), testosterone (B), 17-hydroxyprogesterone (C), or progesterone (D) concentration upon loss of androgen receptor in the adrenal cortex when compared to controls at d80 (one-way ANOVA; n=8, Tukey's post-hoc analysis, error bars SEM).

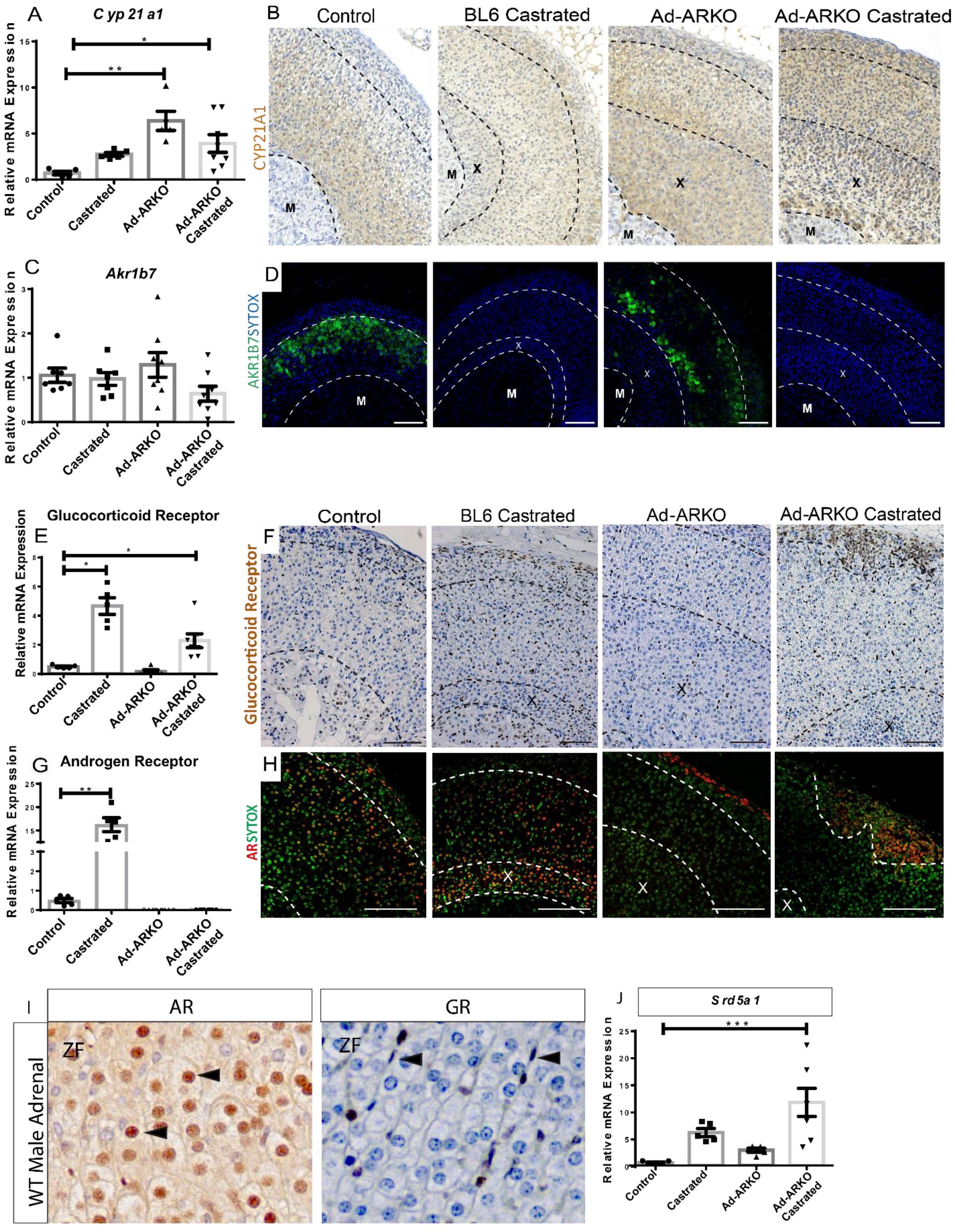

Supplementary Figure 3. Androgens can work independent of their receptor to regulate steroid enzyme activity.

Supplementary Figure 3. Androgens can work independent of their receptor to regulate steroid enzyme activity. (A) Transcript analysis of *Cyp21a1* shows an increase in expression in Ad-ARKO and Ad-ARKO castrated males mice (one-way ANOVA; * $p<0.05$, **$p<0.001$, Tukey's post-hoc analysis, error bars SEM). (B) Interrogation of CYP21A1 protein localisation shows in WT adrenals that staining is localised to the ZF, however upon androgen disruption the staining can be observed in all zones in the cortex. (C) *Ak1b7* gene expression shows no difference in expression any of the cohorts. (D) AKR1B7 immunostaining (Green) is lost in both cohorts that have been castrated, however AKR1B7 expression can still be observed in Ad-ARKO mice. (E) Transcript analysis of GR shows a significant increase in castrated adrenals (one-way ANOVA; n=8, *$P=0.05$, Tukey's post-hoc analysis, error bars SEM) at d80. (F) GR chromogenic immunostaining reveals no changes in localisation through the adrenal cortex at d80 in any cohort analysed. (G) Transcript analysis of androgen receptor shows a significant increase in BL6 castrated adrenals (one-way ANOVA; n=8, $P=0.001$, Tukey's post-hoc analysis, error bars SEM) at d80. (H) AR immunostaining (red) reveals no changes in localisation through the adrenal cortex in control and BL6 castrated cohorts at d80 and as expected is absent in the cortex of both AR ablated cohorts at d80. (I) Chromogenic immunostaining of AR and GR demonstrates localisation of AR and GR in separate cell populations of the zona fasciculata of the adrenal cortex in d80 WT mice. (J) *Srd5a1* gene expression shows a significant increase in expression in Ad-ARKO castrated mice adrenals (one-way ANOVA; n=8 ***$p<0.0001$, error bars SEM) at d80. M=Medulla, ZF= Zona Glomerulosa, ZF= Zona Glomerulosa, X=X-zone. Scale Bars 50μm.

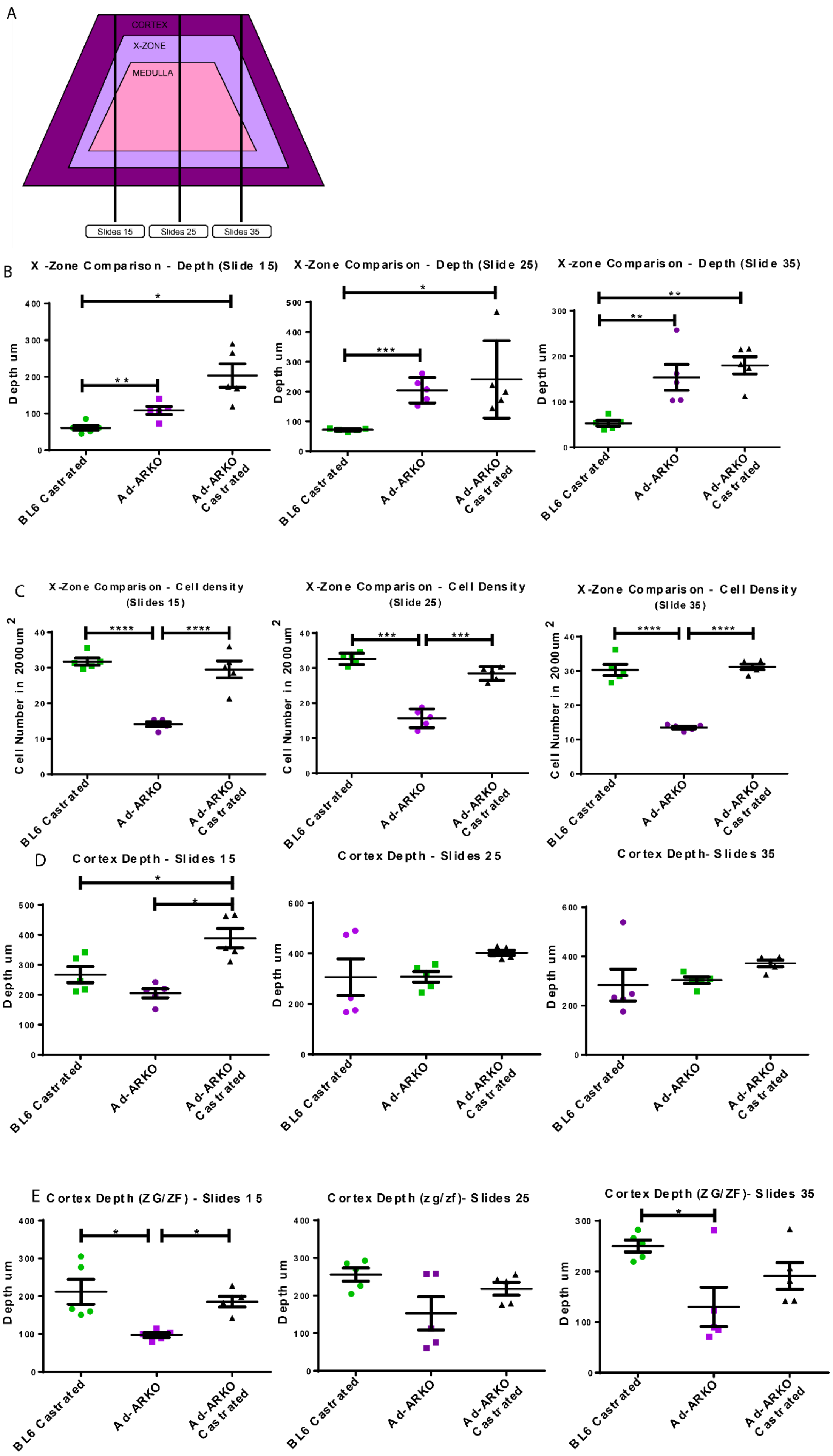

Supplementary Figure 4. Adrenal cortex individual measurements.

Supplementary Figure 4. Adrenal cortex individual measurements. (A) Schematic demonstrating the regions counts and measurements were performed. Measurements were taken at three intervals throughout the cortex at slides 15, 25, and 35. (B) X-zone depth measurement at slides 15, 25 and 35. (B) X-zone depth measurement at slides 15, 25 and 35. (C) X-zone cell density measurement at slides 15, 25 and 35. (D) Overall cortex depth measurements at slides 15, 25 and 35. (one-way ANOVA; n=8, **P=0.05*, Tukey's post-hoc analysis, error bars SEM) (E) Cortex depth of ZG and ZF only measurements at slides 15, 25 and 35 (one-way ANOVA; n=8, **P=0.05*, Tukey's post-hoc analysis, error bars SEM).

	LOQ (nM)	Range (nM)
Estrone 3-sulfate	0.026	LOQ-10
Cortisone	0.19	LOQ-112
Cortisol	1.9	LOQ-794
dehydroepiandrosterone sulfate (DHEAS)	19	LOQ-3000
Corticosterone	0.1	LOQ-144
11-deoxycortisol	0.017	LOQ-40
Δ4-androstenedione	0.042	LOQ-1746
Testosterone	0.012	LOQ-1732
17α-hydroxyprogesterone	0.1	LOQ-1513
Progesterone	0.036	LOQ-500

Supplementary Table 1. LC-MS/MS limits of quantification (LOQ) and range of calibration curves were based on 10 standards prepared as for human serum analysis (60).

	Serum #72 Q Low			Serum #72 Q High			Serum #73 Q Low			Serum #73 Q High		
	mean (nM)	RSD (%)	recovery (%)	mean (nM)	RSD (%)	recovery (%)	mean (nM)	RSD (%)	recovery (%)	mean (nM)	RSD (%)	recovery (%)
Estrone 3-sulfate	0.43	7.0	105	0.94	5.7	99	0.39	8.7	96	0.92	8.9	96
Cortisone	4.63	0.87	100	10.6	7.6	98	4.20	4.1	91	10.4	3.4	97
Cortisol	35.3	8.1	102	81.0	2.3	100	32.3	0.90	93	77.7	1.7	96
dehydroepiandrosterone sulfate	720	7.5	104	1652	2.7	102	671	1.1	97	1584	1.4	98
Corticosterone	38.0	7.3	107	46.5	12	109	18.3	8.7	103	25.5	0.54	98
11-deoxycortisol	1.86	2.3	104	4.07	6.8	98	1.78	1.9	96	4.02	3.4	95
Δ4-androstenedione	2.13	0.74	102	4.72	4.2	97	3.39	6.8	94	5.98	3.0	93
Testosterone	5.62	1.1	111	7.71	3.1	99	49.3	3.0	106	51.4	3.0	98
17α-hydroxyprogesterone	2.09	11	96	4.53	7.0	95	2.18	6.3	90	4.67	0.36	93
Progesterone	3.48	1.3	100	7.99	4.9	98	3.89	5.5	92	8.33	5.2	94

Supplementary Table 2. LC-MS/MS validation: Inter day control materials for this study were prepared in two pools of mouse serum (serum #72 and #73) spiked in low (Q Low) and high (Q High) levels. Results are mean (n=3) of control materials analyzed in three batches.

**Gene**	**Detected?**	***P* value**
Cyp11b1	Yes	0.402
Cyp11b2	Yes	0.8311
Star	Yes	0.8060
Prlr	Yes	0.4013
Pa2g4	Yes	0.7940
Tgfb3	Yes	0.7381
Ctnnb1	Yes	0.4445
Gata6	Yes	0.3521
Wnt4	Yes	0.6777
Gli1	Yes	0.2615
Cyp19a1	No	--
ERα	No	--
ERβ	No	--
Lhr	No	--
Fshr	No	--
Gata4	No	--
Wnt1	No	--

Supplementary Table 3. Genes investigated in this study that revealed no changes in Ad-ARKO mice compared to littermate controls. Genes that showed no detection in adrenal samples had testis, ovary or pituitary controls to ensure that the qPCR had worked.
